# Molecular Genetics in Epstein–Barr Virus-Associated Malignancies

**DOI:** 10.3390/life11070593

**Published:** 2021-06-22

**Authors:** Srikanth Umakanthan, Maryann M Bukelo

**Affiliations:** 1 Pathology Unit, Department of Paraclinical Sciences, Faculty of Medical Sciences, The University of the West Indies, Mount Hope, Trinidad and Tobago; 2 Laboratory Services, Department of Anatomical Pathology, Eric Williams Medical Sciences Complex, North Central Regional Health Authority, Champs Fleurs, Trinidad and Tobago; bukelom@yahoo.com

**Keywords:** Epstein–Barr virus, genome, pathogenesis, lymphomas, carcinomas

## Abstract

Global genomic studies have detected the role of genomic alterations in the pathogenesis of Epstein–Barr virus (EBV)-associated tumors. EBV oncoproteins cause a vital shift of EBV from an infectious virus to an oncogenic form during the latent and lytic phase within the lymphoid B cells and epithelial cells. This epigenetic alteration modulates the virus and host genomes and inactivates and disrupts numerous tumor suppressors and signaling pathways. Genomic profiling has played the main role in identifying EBV cancer pathogenesis and its related targeted therapies. This article reviews the role of genetic changes in EBV-associated lymphomas and carcinomas. This includes the prolific molecular genesis, key diagnostic tools, and target-specific drugs that have been in recent clinical use.

## 1. Introduction

Epstein–Barr virus (EBV) is a lymphotropic virus belonging to the human herpesvirus family. Based on the epidemiological data, EBV infects more than 90% of the world’s population [1]. It was first discovered in the cells of an African patient suffering from Burkitt lymphoma in 1964. The primary infection occurs typically in children and is asymptomatic [2]. As the infection recurs in the adult stage, the infection manifests as infectious mononucleosis. EBV has demonstrated the transformation of resting B cells into lymphoblastoid oncogenic cell lines resulting in the origin of various lymphomas and epithelial malignancies [3].

The genomic variation in EBV promotes the virus transformation into oncogenic cell lines that are responsible for its tumorigenic outcome [4]. Infectious mononucleosis results from the mutagenic variation of HLA-class I, Burkitt lymphoma arises from B cell proliferation due to MYC translocation, Hodgkin’s lymphoma is caused due to the critical role of NF-Kb, EBV produces “trogocytosis” in the development of NK/T cell lymphoma and nasopharyngeal carcinoma occurs due to functional polymorphism of MAP2K4 suppressor gene [5,6,7].

## 2. Material and Methods

A PubMed literature search was performed. We used PubMed’s MeSH terms Epstein–Barr virus (EBV), infectious mononucleosis, lymphoma, carcinoma, lymphoproliferative disorder/cancer, lymph node cancer, and genetics in EBV. Abstracts were screened focusing on the search results relevant to this review. Cochrane reviews, systematic reviews, and meta-analyses were prioritized. Case reports were included as citations and as outliers to the uncommon conditions. Articles focusing on EBV-associated IM, EBV-associated lymphomas, and EBV-associated epithelial malignancies were prioritized. This review search included EBV relevant articles, references, keywords, and the “similar article” tab on the PubMed database. This approach generated 82 relevant research articles including 54 reviews, 23 original articles, and 2 each consisting of book chapters, monograms, and case reports published between 1992 and 2021. Contradicting perspectives, results, and subjective conclusions were scrutinized and referred for relevant original studies, and then presented.

This review provides concise information on the genomic structure of EBV, its infectious cycle, and its progression from infectious phase to malignancies. This work also details the specific genetic pathways and cycles that act as molecular targets during lymphomagenesis and carcinogenesis. The relevance of pharmacological strategies and novel therapeutic options are mentioned, thereby providing a vital link between the molecular genesis and target therapies for EBV-associated malignancies.

## 3. Genomic Structure

EBV genome is composed of 172 kbp linear, double-stranded DNA. The open reading frames (ORF) are involved in encoding proteins for DNA replication, gene expression, and conserving genomic integrity. These coding genes have structural and non-structural functions depending on the ORF sizes [8]. The microRNAs (miRNAs), which include BART and BHRF cluster, are expressed during the various phases of EBV infection and are encoded by the EBV. These miRNAs promote the latent phase of the virus and also target the cellular genes causing carcinomas of various types. The 3′ untranslated region (UTR) in the host genes promotes cancer susceptibility and patient prognosis [9].

Genome sequences reported from previous studies are as follows: (1) B95-8, derived from a case of infectious mononucleosis in North America; (2) WT-EBV, is a more complete reference genome constructed by using B95-8 as its backbone; (3) AG876, derived from a case of Burkitt lymphoma in Western Africa is unique for being the only complete established type 2 genomic sequence of EBV; (4) GD1, (5) GD2, and (6) HKNPC1 are derived from nasopharyngeal carcinoma patients; (7) Akata, derived from Burkitt lymphoma case in Japan; (8) Mutu, derived from a Burkitt lymphoma case in Kenya using NGS technology; (9) C666-1; (10) Raji; (11) K4412-Mi; (12) K4123-Mi; (13) EBVaGC1-9 have also shown regional variations [10,11,12,13].

## 4. Infection Cycle

During the initial stages, EBV spreads through the saliva and affects the tonsils. The naïve B cells and the follicular dendritic cells in the tonsils bind with the surface glycoprotein of the virus through the complement receptor CD21 [14]. This further activates the B cells, and the virus catalyzes the differentiation of the normal cellular pathway of the B cell through the expression of the viral latent proteins: namely, Epstein–Barr nuclear antigens (EBNA) and latent membrane proteins (LMP). Following this differentiation, the B blast enters the germinal center causing down-regulation of the EBNA proteins. This propels the infected B cell into the germinal center, before exiting into the circulating blood as a memory B cell [15]. This latent inactive state is due to the lack of the virus protein expression. Direct infection of either naïve B cells or memory B cells and also marginal zone memory B cells results in the release of terminal differentiation signals triggering a lytic reactivation of the latent virus [16]. This reactivation phase is subdivided into immediate-early, early, and late phases. The infectious virions are now released into saliva for the transmission of the virus to new hosts, thus concluding the infectious cycle [17].

## 5. EBV Associated Human Diseases

EBV primarily causes infectious mononucleosis (IM) and also associated with the development of systemic disorders like rheumatoid arthritis, multiple sclerosis, chronic fatigue syndrome, and Vitamin D deficiency [18]. Malignancies caused by EBV include Burkitt lymphoma, Hodgkin’s lymphoma, NK/T-cell lymphoma, nasopharyngeal carcinoma, gastric carcinoma, and breast carcinoma [19]. The genetic origin of these individual lesions along with diagnostic and treatment aspects are reviewed. Since IM forms the basis of EBV oncogenic transformation, IM is included in this review along with other malignancies.

## 6. Infectious Mononucleosis (IM)

IM is predominantly caused by EBV through the saliva of an infected person and is transmitted by coughing and kissing. It commonly affects adolescents and young adults [20]. EBV viral concentration is detected in the saliva at a wide range of concentrations, peaking during the acute phase. EBV infects the oropharyngeal epithelial cells and the resting B-cells, following which the virus replication is initiated. This period is devoted to incubation, lasting from four to eight weeks. The next phase involves activation of cytotoxic T-lymphocytes and the natural killer cells, inducing a cell-mediated immunity [21]. Reactivation can occur during the later period of life elicited by host immunosuppression events such as infections (e.g., HIV), medications, and surgical transplants [22].

EBV-induced host genetic variation has been highlighted by many gene studies. IM- associated genomic regions and polymorphism of HLA-class I have a higher risk of developing IM. Recent studies have further included homozygous allele 1 of STR D6S510 and STR D6S265 in the high-risk list for developing IM [21,23]. Genomic sequencing has identified certain genetic disorders that are susceptible to develop EBV-induced IM. These include CORO1A, XMEN, ITK, and PRKCD deficiency. CORO1A deficiency causes primary immunodeficiency, XMEN deficiency leads to general immunodeficiency, ITK deficiency causes fatal B-cell proliferation, and PRKCD causes autoimmune lymphoproliferative syndrome [21,22,24].

Clinical features include fever, pharyngitis, cervical lymphadenopathy, tonsillitis, and palatal petechiae. Laboratory evaluation shows lymphocytosis and elevated alanine aminotransferase [25]. 85% of EBV infected patients show a positive Monospot test (heterophile antibody test): a famous laboratory test, owing to its rapid diagnosis and low cost. VCA IgM antibody is specific during the early infectious period and VCA IgG antibody is best for past infection diagnosis. Both these tests are not usually performed at the point of care sites [26,27]. Treatment for IM mainly focuses on symptomatic relief by rest, fluid replacement therapy, antipyretics, and analgesics. Antiviral drugs like acyclovir, valacyclovir, and steroid usage have been of limited clinical benefit [28].

## 7. Burkitt Lymphoma (BL)

BL is classified into endemic, sporadic, and HIV-associated types. EBV is associated with over 90% of endemic BL and is prevalent in regions where malaria is hyper-endemic such as in the equatorial African regions [29]. HIV-associated types are EBV positive in approximately 30%. Endemic EBV-associated BL is common in children and often presents with involvement of the jaw, facial bones, renal, and extranodal sites [30].

The B cells infected by the EBV along with MYC translocation and co-factors such as malaria and HIV infection initiates B cell activation and proliferation. As the number of B cell infections rises, there is germinal center expansion and accumulation of oncogenic mutation [31]. The coupling effect of malaria-mediated activation and germinal center B cell proliferation results in the formation of BL progenitor cells. The vital proliferative and apoptotic pathways include gene activating and gene inhibitory mutations. The gene activating mutations are the c-MYC, TCF3, and CCND3. The gene inhibitory mutations include P53, PTEN, ID3, and CDKN2A [32,33].

**Figure 1:**
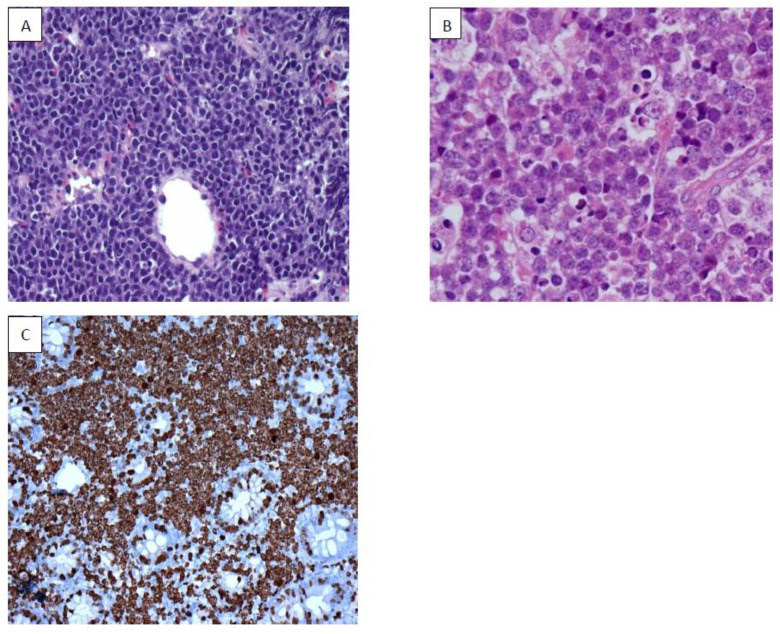
Burkitt Lymphoma: (**A**) Diffuse effacement of the nodal architecture by atypical lymphoid cells. (**B**) Starry sky pattern (**C**) high Mib-1 labeling index.

Histologically this tumor type exhibits diffuse effacement of the nodal architecture replaced by medium-sized monomorphic tumor cells with round nuclei and numerous nucleoli. Due to their high proliferation rate, presence of numerous atypical mitosis, macrophages, and apoptotic debris, they display a striking “starry sky” pattern (Figure 1A,B). The diagnosis of BL is dependent solely on its genetic and immunophenotype analysis such as CD10, CD 20, and high Mib-1 labeling index (Figure 1C) [31,34].

However, imaging studies contribute to the evaluation of the clinical course, treatment response, and complications of BL [35]. This aggressive lymphoma is treated with multi-drug chemotherapy and immunotherapy. The ID3/EZA/cyclinD3 pathway targeted therapy and inhibitors of the c-MYC, PI3 kinase and Bcl2 family are under detailed genetic mapping for the development of new therapeutic target agents [36].

## 8. Hodgkin’s Lymphoma (HL)

Hodgkin’s disease was first described by Sir Thomas Hodgkin in 1832. Since then, there has been controversy on whether to classify it as an inflammatory, infectious, or malignant disease [37]. HL has shown typical bimodal age distribution with EBV+ cases mainly showing an initial peak in children of developing nations. The geographic distribution of EBV+HL cases is across Kenya (92%), China (65%), South and Central America (50–95%), and North America and Europe (20–50%) [38].

EBV infection targets the naïve B cells, interferes with B cell genetic differentiation, and rescues B-cell receptor-deficient germinal center B cells from apoptosis. NF-kB plays a critical role during the rescue process by activating the expression of anti-apoptotic DISC-inhibitor c-FLIP [39]. These EBV-infected HL cells express various LMPs that activate NF-kB. Notch ligation has proven to play a vital role in LMP-1 regulation by inhibiting LMP-1 expression and promoting EBV nuclear antigen -2 (EBNA2) during the primary infectious phase [38,40]. Activated Notch ligation also downregulates LMP2A during the lymphoblastoid transformation of B cells. LMP-1 promotes the expression of the collagen receptor, discoidin domain receptor 1 (DDR 1) that forms a major chronic inflammatory component for the HL microenvironment. LMP-1 expression explains the varied presentation of EBV-infected patients from an asymptomatic phase to a severe potent oncogenic phase. In some HL cases, EBV causes inactivation of A20 tumor suppressor gene alleles [39,41,42].

**Figure 2:**
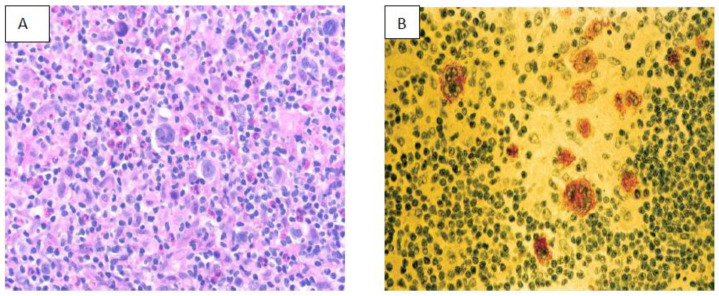
Hodgkin’s lymphoma (**A**) in HIV (**B**) positive immunostain for Epstein–Barr virus-latent membrane protein.

HIV- infected patients almost always express type II latency of EBV infection (Figure 2A,B). This is characterized by morphological differences by increased CD 163+ spindle-shaped macrophages forming a “sarcomatoid” pattern. This provides a favorable treatment using the cART regime (doxorubicin, bleomycin, vinblastine, dacarbazine) providing an outcome similar to HIV-negative patients [43,44].

## 9. NK/T Cell Lymphoma (NKTCL)

EBV is well documented as an oncogenic virus in B cell neoplasm; however, its role in the pathogenesis of NKTCL has been complex. Mature NKTCL is a group of heterogeneous tumors that have complex treatment options, aggressive clinical presentation, and poor prognosis [45]. The geographic distribution of EBV-induced NKTCL has been more concentrated in Asia (China, Korea), South America (Peru, Argentina), Central America (Mexico), and Central Africa. EBV in NKTCL is classified into nodal and extra-nodal types. The role of EBV in angioimmunoblastic T-cell lymphoma, nasal type of extranodal NKTL, and aggressive NK- cell leukemia is well researched and documented [46].

The role of EBV infection in NKTCL has been hypothesized during the targeted killing of EBV-infected cells by NK/T cells. The patients with overlapping evidence of EBV- associated diseases are shown to have circulating EBV-infected NK/T cells (EBV positive NK/T cells) [46,47]. This positivity is due to the effect of the “trogocytosis” phenomenon caused by glycoproteins (gp 350, gp 42, and gp85) and cellular protein (CD 21). This further induces the expression of EBNA (types 1,2,3A,3B,3C) and LMP (1,2A,2B) proteins that result in genetic polymorphisims. LMP1 acts as an active member of tumor necrosis factor (TNF) and downregulates NF-Kb and MAPK signal pathways [45,46,48]. This further causes variation in the C-terminus of LMP1, deletion of 30bp, 33bp repeats, insertion of 15bp, and other amino acid substitutions. These genetic expressions, deletions, and variations have been studied in numerous molecular genetic pathways using whole-genome sequencing, targeted and exome sequencing. This has allowed the discovery of more target-specific therapies to these sets of complex malignancies [46,49]. The most common genetic alteration in EBV-induced NKTCL is the loss of 6q21 (20–43% cases) followed by recurrent losses in chromosomes 1p4 and 5p13. JAK-STAT signaling pathways along with STAT3, KMTZD, and TP53 are the most recurrently mutated genes in EBV-induced NKTCL [50].

Extranodal NKTCL occurs predominantly in midline facial structures and can also affect paranasal sinuses, orbit, jaw, and salivary glands. Symptoms vary from systemic features (fever, weight loss, night sweats) to hemophagocytic disorders [51]. Imaging studies (CT, MRI, PET/CT) coupled with EBV-DNA biomarker assessment are essential for diagnosis [52].

Treatment modality varies on the stage of NKTCL. Stage I/II are treated mainly with radiotherapy. Previous chemotherapeutic regimes using anthracycline-based CHOP (cyclophosphamide, doxorubicin, vincristine, and prednisone) have shown to cause more toxicity and drug resistance [53]. The recently introduced LVP (L-asparaginase, vincristine, and prednisone) and GELOX (gemcitabine, oxaliplatin, and L-asparaginase) are shown to be more effective in this stage [51,54]. Advanced/relapsed/recurrent NKTCL are treated with selective chemotherapy but due to poor host response and drug-induced toxicity has resulted in poor patient prognosis in many of the cases. Hematopoietic stem cell transplant and immunotherapy are still under trial [55].

## 10. Nasopharyngeal Carcinoma (NPC)

NPC is commonly seen in the fossa of Rosenmuller. This malignant tumor of the nasopharynx has a striking geographic distribution with a predilection to the Southeast Asian and Northern African populations [56]. They are classified histologically into keratinizing (type I) and non-keratinizing types. The non-keratinizing type is further classified into differentiated (type II) and undifferentiated (type III) variants. EBV belongs to group 1 carcinogenic causative agent for type III NPC [57]. Once the EBV infects the squamous epithelial cell, the clonal proliferation of these EBV-infected cells expresses LMP-1, LMP-2, and EBNA-1 as gene products. LMP-1 plays a key role in NPC tumorigenesis. It is involved in B-cell transformation, induction of surface adhesion molecules, and enhancement of anti-apoptotic Bcl-1 proteins, and production of IL-6 and IL-8. EBNA 1 plays a vital role in tumor cell segregation, replication, and resisting apoptosis [58,59].

EBV transmission in humans during the infectious cycle depends on the involvement of the epithelial cells and the B lymphocytes. B lymphocytes are easily infected and transformed into atypical lymphoblastoid cells followed by the immortalizing phase [60]. In the epithelial cells of the pharynx, they persist as reservoirs of the EBV genomes which are eventually released for transmission through saliva [58,59,61].

A genetic functional polymorphism in a candidate suppressor gene, MAP2K4 has been found in almost 90% of NPC patients. Genome-wide association analysis has found statistically significant frequencies in HLA-A (02:07), HLA-B (27:04, 46:01, and 58:01) and HLA-C (01:02, 03:02, and 12:02) alleles [57,62]. NPC cells that are EBV-encoded (miRNAs, miR-BARTs) facilitate EBV latency, accelerate cellular tumor proliferation, induce genomic mutation and impair host immune repair function. LMP1 forms a key EBV-oncoprotein to generate NPC tumorigenesis by activating NF-Kb, MAPK, JNK/AP1, and PI3K signal pathways, transforming nasopharyngeal epithelial cells into cancer cells [63].

Factors that contribute to the development of EBV-induced NPC include childhood exposure to EBV infection. Childhood family structure, order of birth, poor oral hygiene, familial aggregation, and genetic susceptibility such as HLA-A2-B46 and B17 are all linked to a higher risk of developing EBV-related NPC [64]. A combination of IgA antibodies against the Epstein–Barr nuclear antigen 1 (EBNA1/IgA) and VCA/IgA measured by enzyme-linked immunosorbent assay (ELISA) is the most sensitive and specific detecting method [65].

## 11. EBV-Associated Gastric Ca (EBVaGCs)

EBVaGCs constitute approximately 10% of the overhaul gastric carcinomas in the world with approximately 950,000 cases [66]. The variation in its incidence across the globe is well documented with the clustering of cases in Europe, South East Asia, and the Americas [67].

Gastric carcinomas are classified into four molecular subtypes: EBVaGC, microsatellite instability, chromosomal instability, and genomically stable tumors [66,68]. PIK3CA mutations acquired through EBV cause host DNA hyper-methylation causing intestinal-type gastric carcinomas in the lower third of the stomach. Hematogenous spread and upper third gastric carcinoma were also associated with PIK3CA mutations [69]. The binding between H. pylori tyrosine-phosphorylated cytotoxin-associated gene A (Cag A) and tyrosine phosphatase SHP2 of EBV results in deregulation of the phosphatase activity. This binding-interaction mechanism between H. pylori and EBV has been considered to play an important role in gastric carcinogenesis. EBV infection can promote methylation of SHP1, thereby increasing the oncogenic function caused by the interaction of Cag A and SHP2 [68,70,71]. Recent molecular studies have shown that EHF is a critical factor to enhance and promote tumor cell proliferation in EBVaGC. The upregulation of EHF is caused by LMP2A (EBV protein) via STAT3 phosphorylation [72]. CXCL11 was found to be a prognostic biomarker and also measured the extent of tumor progression [73].

EBVaGC is predominant in males and smokers. The lesions are either ulcerated or fungating in nature. The submucosal tumor-like morphology is featured as a hypoechoic nodule on endosonography [74]. The histomorphology reveals tumor cells and lymphocytes, owing to its carcinogenesis. The histologic subtypes of EBVaGC are lymphoepithelioma-like carcinoma (LELC)-type, conventional type adenocarcinoma (CA)-type, and carcinoma Crohn’s disease- like lymphoid reaction (CLR)-type [75].

Studies have documented that EBVaGC has a lower lymph node metastasis compared to EBV-negative gastric carcinomas more so during the early stages of EBVaGC [76]. This along with a higher rate of endoscopic detection of the early EBVaGC, they have a favorable prognosis and longer survival rates compared with EBV-negative gastric carcinomas [77].

## 12. EBV Related Breast Cancer (EBVrBCa)

Breast cancer (BCa) is the most common malignancy affecting females globally [78]. The etiology is mainly related to genetically inherited factors including BRCA 1, BRCA 2, and HER-2 [79]. Non-genetic factors include infection with viral agents such as HPV, CMV, and EBV. EBVrBCa has been documented in Africa, India, China, and Europe [80]. EBV primarily infects the mammary epithelial cells (MEC’s) and CD21. This further causes expansion of the progenitor stem cell phenotypes resulting in blockage of cell differentiation. These pathways along with Ras activation promote the development of BCa [81]. EBVrBCa has a high proliferative rate (Ki67), is basal type, and negative for ER, PR, and HER-2neu [78,82]. These tumors are positive for CK 5/6, CK 14, and vimentin [83]. EBVrBCa is more common in younger females, is present with a larger tumor size, and has a higher histological grade, thereby it is associated with lower overall survival rates [79,84].

## 13. Genetic Targeted Pharmacological Strategies in EBV Associated Malignancies

Cell cycle dysregulation forms a vital role in EBV-mediated oncogenesis. Cyclin D1 and cyclin D2 proteins present in EBV-transformed cells facilitate G1/S transition and uncontrolled cellular proliferation [85,86]. The cyclin D activity is regulated by CDK inhibitory proteins, thereby providing CDK inhibitors as potential targets in restricting the transformation of the latent phase to a lytic infection during EBV oncogenesis. EBV infection induces dysregulation of PI3K pathway, MAPK pathway, JAK/STAT pathway, and NF-Kb pathway during lymphomagenesis. These pathways and the involved molecules serve as potential therapeutic targets in EBV-induced lymphomas [87].

EBV also utilizes several mechanisms that block apoptosis and activate oncogenesis through Bcl-2 (anti-apoptotic protein) and Bcl-6 (master regulator of germinal center B-cell development) during lymphomagenesis [88]. These can be targeted by using selective inhibitors such as ABT-199 and BHRF-1 that act on Bcl-2 and block apoptosis and suppress tumor growth in EBV-positive B-cell lymphomas [89]. Ixazomib and bortezomib are proteasome inhibitors that act by inducing apoptosis and also cause cell cycle arrest in EBV-associated lymphomas.

New emerging therapeutic techniques include proteolysis targeting chimeras (PROTAC) and hydrophobic tagging (HyT) technique [90,91]. These can accelerate the development of anti-EBV drugs. Immune checkpoint inhibitors (PD-1/PD-L1 antibody), monoclonal antibodies (anti-CD20 antibody rituximab), and chimeric antigen receptor (CAR)-modified T-cell therapy are the future potential therapies targeting EBV associated malignancies [87,92].

## 14. Conclusions

The study of genomic integration of EBV into the host human genome resulting in instability and disruption of gene expression has been the main objective of various molecular research institutes that focus on EBV study. The unique nature of EBV to express gene heterogeneities and phenotypic changes has led to challenges in tapping its oncogenic nature and identifying a possible pathogenic pathway. Recent research has identified the high-risk groups of people who are vulnerable to EBV’s, who can then be target diagnosed and treated for these life-threatening cancers. Nevertheless, EBV vaccine development solely depends on these noble research studies.

## Data Availability

Not applicable.
